# Mineral Surface Chemistry and Nanoparticle-aggregation Control Membrane Self-Assembly

**DOI:** 10.1038/srep43418

**Published:** 2017-03-07

**Authors:** Nita Sahai, Hussein Kaddour, Punam Dalai, Ziqiu Wang, Garrett Bass, Min Gao

**Affiliations:** 1 Department of Polymer Science, University of Akron, Akron, OH 44325, USA; 2 Department of Geology, University of Akron, Akron, OH 44325, USA; 3 Integrated Bioscience Program, University of Akron, Akron, OH 44325, USA; 4 Liquid Crystal Institute, Kent State University, Kent, OH 44240, USA.

## Abstract

The self-assembly of lipid bilayer membranes to enclose functional biomolecules, thus defining a “protocell,” was a seminal moment in the emergence of life on Earth and likely occurred at the micro-environment of the mineral-water interface. Mineral-lipid interactions are also relevant in biomedical, industrial and technological processes. Yet, no structure-activity relationships (SARs) have been identified to predict lipid self-assembly at mineral surfaces. Here we examined the influence of minerals on the self-assembly and survival of vesicles composed of single chain amphiphiles as model protocell membranes. The apparent critical vesicle concentration (CVC) increased in the presence of positively-charged nanoparticulate minerals at high loadings (mg/mL) suggesting unfavorable membrane self-assembly in such situations. Above the CVC, initial vesicle formation rates were faster in the presence of minerals. Rates were correlated with the mineral’s isoelectric point (IEP) and reactive surface area. The IEP depends on the crystal structure, chemical composition and surface hydration. Thus, membrane self-assembly showed rational dependence on fundamental mineral properties. Once formed, membrane permeability (integrity) was unaffected by minerals. Suggesting that, protocells could have survived on rock surfaces. These SARs may help predict the formation and survival of protocell membranes on early Earth and other rocky planets, and amphiphile-mineral interactions in diverse other phenomena.

In modern geochemical environments, orders of magnitude greater numbers of prokaryotic cells live in biofilm communities at the sediment-water interface than in the water column, because of the numerous metabolic and protective benefits provided by such interfaces^1^,^2^,^3^. Even planktonic cells are brought in contact with suspended sediments in environments such as beaches where the kinetic energy provided by waves and currents can re-suspend sediments. Planktonic cells also come in contact with bottom sediments during wetting-drying cycles of intertidal pools and inland salt lake basins. It is reasonable, then, to assume that primitive “protocells” on early Earth would have been in contact with the mineral-water interface. A natural question that follows is whether the properties of minerals and rocks could have influenced the formation of lipid membranes, which formed the boundary between the protocell and its environment. Single chain amphiphiles (SCAs), mainly fatty acids and fatty alcohols, have been employed extensively in the literature as model protocell membranes in origins of life studies^4^,^5^,^6^,^7^,^8^ because it is believed that abiotic phospholipid synthesis would have been too difficult. Also, SCAs have been isolated from carbonaceous chondrites and synthesized under hydrothermal conditions, which suggests that they may have been present on early Earth^9^,^10^,^11^,^12^. Inorganic solid-lipid interactions are also important in a diverse array of fields, for example, in petroleum recovery, drug delivery, improving biocompatibility of biomaterial surfaces by deposition of phospholipid bilayers, clean energy applications and cytotoxicity of nanoparticles to human and bacterial cells.

The potential effect of minerals on the rate of formation of SCA vesicles as model protocell membranes has been examined previously^13^,^14^. In particular, the rate of phase transition from micelles to vesicles was studied. All the minerals studied enhanced the initial micelle-to-vesicle phase transition rate compared to systems without minerals. It was proposed that vesicle formation is promoted close to the mineral surface but not in direct contact with the surface. All the minerals assisted vesicle formation but no relationship with chemical, structural or physical properties of the minerals was identified. Furthermore, no systematic trends are known between mineral properties and other prebiotic processes such as potential effects of minerals on the thermodynamics of SCA self-assembly (*i.e.*, CVC), membrane stability (permeability), mineral-enhanced nucleotide polymerization or photocatalytic-mineral mediated proto metabolism^15^,^16^,^17^.

On the other hand, the stability and formation kinetics of phospholipid bilayer membranes as well as the adhesion of prokaryotic and eukaryotic cells to oxide and aluminosilicate (muscovite mica) mineral surfaces is known to depend on the surface charge of minerals^18^,^19^,^20^,^21^,^22^,^23^. Surface charge depends on the isoelectric point (IEP) of the mineral. Adhesion of phospholipid bilayers, bacteria and T-lymphocytes is driven by a combination of electrostatic, van der Waals and H-bonding interactions (and, additionally, by covalent bonds in the case of T-lymphocytes). It is proposed here that similar physical interactions should control the association of SCAs with mineral surfaces. The goals of the present study were: (i) to examine the potential effects of minerals on the thermodynamics and kinetics of initial vesicle self-assembly and survival after formation, and (ii) to identify mineral-specific trends in these processes, if any. The thermodynamics of vesicle self-assembly were estimated by determining CVC. Kinetics of vesicle self-assembly were determined for *de novo* vesicle formation by lipid thin film rehydration and for the phase transition of micelles to vesicles. After vesicles are formed, their survival was estimated by quantitating membrane permeability integrity over time. All experiments were conducted in the presence and absence of minerals. In order to identify mineral-specific trends, diverse potentially complicating factors were accounted for including accurate characterization of the multiple bulk and surface properties of minerals; partial dissolution of some minerals with the formation of secondary minerals, which can alter the original surface; and gravitational settling of particles, which may limit contact with vesicles.

In the present study, the SCAs, lipids, rocks and minerals were chosen to represent those that would likely have been present on early Earth. Decanoic acid (DA, apparent pK_a_ ~7–7.2^24^) and mixed DA/decanol (DOH) systems were chosen as the model membrane-forming SCAs. The relatively short chain amphiphiles (C8–C10) have been used in several other studies involving vesicles^6^,^25^,^26^,^27^. Such SCAs form more unstable and leaky vesicles than longer chain amphiphiles, so they are difficult to work with; however they are more plausible prebiotically, because the SCAs extracted from meteorites are also of shorter chain lengths^28^,^29^ (up to C9). Myristoleic acid (C14), palmitic acid (C16) and oleic acid (C18 and one unsaturated C=C bond) were used in a previous study of micelle-vesicle phase transition kinetics in the presence of minerals^14^. The rocks and minerals used in the present study were chosen to represent the crustal rocks on Hadean and Eoarchean Earth, and the minerals produced by weathering these rocks under atmospheric conditions of high partial pressure of CO_2_, presence of H_2_S and lack of molecular O_2_^11^,^30^,^31^. In detail, komatiite and tonalite formed the earliest oceanic and proto-continental crust, respectively, as early as ~4.4–3.8 Ga^32^,^33^. The minerals chosen for the present study were amorphous silica (amorphous SiO_2_), quartz (α-SiO_2_), montmorillonite (Na_0.2_Ca_0.1_Al_2_Si_4_O_10_(OH)_2_(H_2_O)_10_), rutile (α-TiO_2_), anatase (β-SiO_2_), pyrite (FeS_2_), siderite (FeCO_3_, idealized stoichiometry), zincite (ZnO), γ-alumina (Al_2_O_3_) and goethite (α-FeOOH). Calcium, sodium, silica and alumina released by dissolution of komatiite and tonalite would have formed montmorillonite, chalcedony and boehmite. amorphous SiO_2_ and γ-Al_2_O_3_ are model analogs here for chalcedony and boehmite. Pyrite and siderite would have formed from the ferrous iron released by dissolution of komatiite and released at hydrothermal vents into solutions containing dissolved HCO_3_^−^ and HS^−^ ions. Rutile and anatase would have been present in small quantities, and ZnO would have been rare. These latter three minerals are model oxides chosen to represent a range of IEP of the minerals. Goethite is included for reasons explained below. These rocks and minerals also encompass a wide range of chemical compositions including oxides, oxyhydroxide, aluminosilicates, carbonate and sulfide. All solids will subsequently be referred to, *sensu lato*, as “minerals”.

Experimental conditions (solution pH and mineral particle loadings, mg.mL^−1^) were designed to represent two geological scenarios. The rate of micelle-to-vesicle phase transition was monitored as pH was decreased from highly alkaline (pH~12) to neutral (see Methods). This is a very rapid reaction (~minutes) and attempts to mimic the rapid mixing of solutions with different pHs. The system represents the reaction of ocean water with komatiite or with peridotite, which comprises the rock of the upper mantle. This serpentinization reaction produces very alkaline water and could be followed by mixing with ambient seawater, which was slightly acidic because of high CO_2_ content of the early atmosphere. The final solution pH would have been near neutral. Acidic and basic mixing zones may also have existed at the groundwater-vadose water interface in the terrestrial subsurface region^31^. Decanoic acid was used for the micelle-to-vesicle phase transition reaction. The second model system represented terrestrial environments that undergo wetting-drying events in a sedimentary basin under slightly alkaline conditions. Vesicle formation was modeled by rehydration of DA/DOH thin films over a period of several hours (Methods). In both types of model systems, experiments were conducted at various particle loadings to represent a range of sediment loads in low- and high-kinetic energy environments. Low-energy environments have still waters in which particles settle out resulting in a low sediment load, *e.g.*, lake-water on a day without wind. High-energy environments are capable of carrying high sediment loads because of the kinetic energy provided by waves, currents and tides, *e.g.*, flooded rivers and ocean beaches. In some environments such as glacial lakes, the kinetic energy may be low but the sediment load may still be high. This is possible because nanoparticles are created by the grinding action of glaciers on the rocks over which they pass, and these nanoparticles can remain in suspension in relatively pure glacial melt-water where dissolved salt concentrations are very low.

## Results and Discussion

### Mineral Characterization

Mineral properties were characterized in detail (Table 1; Methods; Extended Data Figure 1). The IEPs of the minerals span a wide range from ~2 for amorphous SiO_2_ to 9.3 for γ-Al_2_O_3_. The value for each mineral is represented within a small range of less than one pH unit.

The approximate size of the individual particles as well as of clusters of particles were estimated from transmission electron microscopy bright field images to range from 10 s of nanometers to 5 μm, depending on the mineral (Extended Data Figure 1). The individual, discrete grains of amorphous SiO_2_ and anatase appear sub-spherical and rhombohedral, respectively, of diameter ~10–15 nm. Goethite and γ-Al_2_O_3_ particles are more cylindrical in shape with diameters of ~10–15 nm and length ~200 nm, reflecting the hexagonal prism crystal form. All four phases form clusters or aggregates that are ~50–500 nm in size. The other minerals show substantially larger individual grains of sizes above ~200–300 nm and even as large as a few microns. Among the larger minerals, rutile and ZnO possess well-developed, smooth crystal facets of hexagonal prism and tetragonal crystal forms, respectively. Komatiite, tonalite, siderite and pyrite particles show sharp edges reflecting the crushing process during sample preparation.

The specific surface area of the minerals was determined by multipoint B.E.T. N_2_ gas adsorption isotherm (Methods). The values vary widely from as low as 0.5 m^2^.g^−1^ for pyrite to 119 and 288 m^2^.g^−1^, respectively, for γ-Al_2_O_3_ and amorphous silica.

### Mineral-Vesicle Association

**Figure 1:**
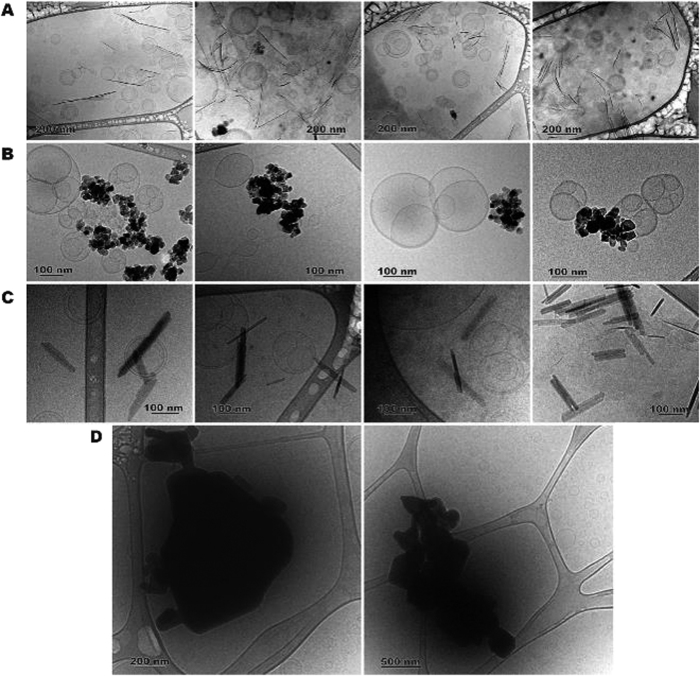
Mineral-vesicle interaction imaged by cryo-TEM. Electron dense areas are the minerals (**A**) montmorillonite; (**B**) anatase; (**C**) goethite and (**D**) zincite. Suspensions were prepared by mixing 5 mg.mL^−1^ mineral in HEPES buffer (pH 7.1) with 75 mM DA micelles (100 mM for zincite). The pH was adjusted to 7.8 with NaOH and the suspension was interacted overnight on a rotor/mixer. Vesicle sizes range from 50 to 1000 nm.

Mineral-vesicle association was visualized by cryo-Transmission Electron Microscopy (Fig. 1; Methods). Minerals appear as dark, electron-dense features; less electron dense, spherical or sub-spherical structures varying in size from ~50 or 80 nm to ~500 nm are interpreted as vesicles (Fig. 1). Unilamellar and multilamellar vesicles can be seen occurring as separate entities or in groups. The curvilinear, dark features about 200–300 nm long in Fig. 1A are montmorillonite clay platelets viewed edge-on. The crystals appear as small hexagonal plates when observed face-on, for example, in the middle and bottom left areas of the second panel of Fig. 1A. Anatase rhombohedral, goethite prismatic cylinders and zincite tetrahedral crystals are seen in Fig. 1B–D. The minerals are directly in contact or in close association with vesicles in all cases.

Minerals and lipids were also visualized by phase contrast microscopy (Methods; data not shown). In buffer solution alone, minerals appeared either as large, discrete particles or as small clusters, especially notable for the nanoparticulate minerals (amorphous silica, anatase and γ-Al_2_O_3_). In the presence of DA or DA/DOH, the minerals and lipids formed larger aggregates indicating the association of lipid with mineral surfaces but vesicles were abundant and dominated the field of view in all cases.

### Vesicle Formation Rates

**Figure 2:**
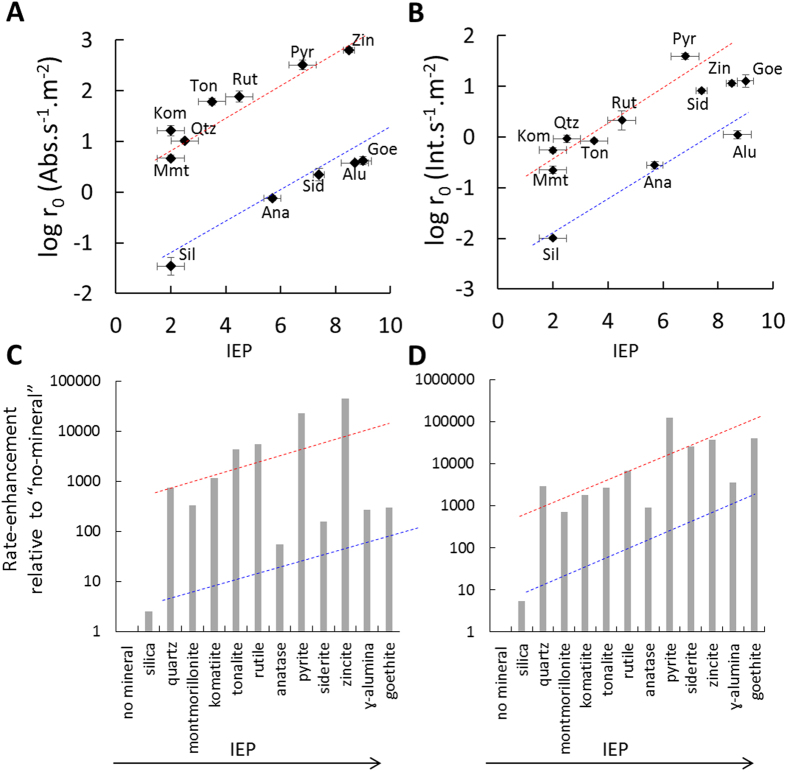
Initial rates of vesicle formation normalized to the mineral surface area (r_0_) as a function of IEP of the minerals. (**A**) DA vesicle formation from micelles at final pH 7.1 ± 0.1; (**B**) DA/DOH vesicle formation from rehydrating lipid thin films at pH 8.1 ± 0.2. Error bars represent the standard deviation of the slope calculated using the LINEST function of excel 2013 (see Methods and Extended Data Figures 3 and 4 for details). (**C**,**D**) Extent of initial rate enhancement per m^2^ of a mineral as compared to the no-mineral control. The dashed blue and red lines, respectively, indicate the trends for the nano and micro-meter sized particle.

**Figure 3:**
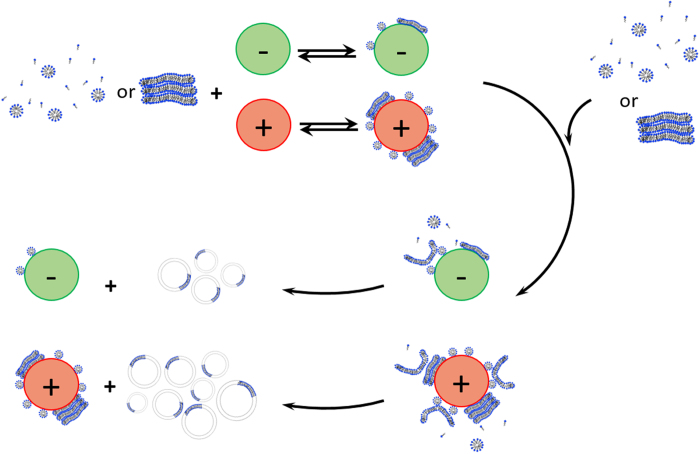
Schematic of the proposed model for mineral enhancing rate of vesicle formation. In the first step, bilayers formed by rehydration of lipid thin films or micelles rapidly adsorb on mineral surfaces forming islands that partially coat the mineral. A greater amount of adsorption of negatively-charged lipids occurs on positively-charged mineral than on negatively-charged minerals. In the second step, the lipid islands partially-coating the mineral act as a template for further attachment of lipid from solution and self-assemble to form vesicles. Thus, the lipid islands catalyze vesicle formation. The more the adsorbed lipid, the greater is the observed rate-enhancement effect. The adsorbed islands may stack up several nanometers away from the surface.

The increase in UV-Vis absorbance (turbidity) and fluorescence intensity of a membrane-soluble dye over time were used to estimate vesicle formation rates in two geological environments as described above (Methods; Extended Data Figure 2). These methods have been used previously as estimates of vesicle formation in the absence of minerals^14^,^34^,^35^,^36^,^37^. All minerals enhanced initial vesicle formation rates relative to the control system without minerals (Fig. 2 and Extended Data Figures 3 and 4). This enhancement was found in both model environments despite significant differences in the experimental set up and duration. The initial rate-enhancing effect may be interpreted as a result of rapid lipid adsorption on mineral surfaces. The adsorbed lipid islands on the partially-coated mineral then serve as a template for more lipid to attach from solution and rapidly transform to vesicles compared to the relatively slower micelle-micelle interactions forming vesicles in the absence of minerals (Fig. 3). A similar mechanism has been proposed earlier where it was suggested that fatty acid vesicle formation is catalyzed close to mineral particle surfaces, but without necessarily requiring lipid adsorption or direct contact of fatty acid with the mineral surface^14^. The catalytic effect of lipid aggregate surfaces in micelle-to-vesicle phase transition and vesicle growth without minerals present has also been noted previously and has been termed the “matrix effect”^34^,^35^,^36^,^38^,^39^.

We found that the initial vesicle formation rates were strongly correlated with the IEP of the minerals for both DA and DA/DOH (2:1) systems (Fig. 2). In our proposed model, the adsorbed lipid islands provide the template or “matrix” for rapid initial vesicle formation. Both DA and DA/DOH micelles carry a net negative charge. Hence, more lipid should adsorb on positively-charged mineral surfaces and a greater rate-enhancing templation effect is expected (Fig. 3). Minerals possess a net positive or negative surface charge because of protonation and deprotonation of surface hydroxyl sites (>MOH°) resulting, respectively, in the formation of positively-charged (>MOH_2_^+^) and negatively-charged sites (>MO^−^) (Extended Data Figure 5A). The negatively-charged DA head groups associate with positively-charged mineral surface sites due to electrostatic interactions. Interestingly, minerals with net negatively-charged surfaces also enhanced the rates of vesicle formation despite expected electrostatic repulsion with both DA and DA/DOH. This effect can be explained by recognizing that, even when minerals possess a net charge, the majority of surface sites are neutrally-charged over a wide range of pHs^40^ (>MOH° = ~75–80%) (Extended Data Figure 5B). Hence, some adsorption occurs because of hydrogen bonding between the fraction of neutrally-charged mineral surface sites and negatively-charged DA head groups (>MOH°---DA^−^) as well as between the negatively-charged mineral surface sites and the fraction of neutral DA and DOH head groups (>MO^−^---DA° or >MO^−^---DOH°). According to the Derjaguin-Landau-Verwey-Overbeek (DLVO) theory for colloid-scale interactions, Van der Waals forces between the lipid and the mineral as well as between adjacent lipid layers also contribute to SCA association with mineral surfaces. The proposed adsorption of lipids by H-bonding and van der Waals forces was supported by the observation that both DA and DA/DOH adsorb on silica (Extended Data Figure 5C,D; Methods) despite expected repulsion between these negatively- charged lipids and the negatively-charged silica surface. Furthermore, the effect of electrostatic forces is reflected in the ~6–10 times greater adsorption on alumina (positively-charged) than on silica (Extended Data Figure 5C,D).

The IEP of a mineral is related to its surface acidity equilibrium constants and can be predicted quantitatively based on consideration of two main contributions to the total protonation free energy, namely the interfacial solvation energy and the energy terms arising from the crystal chemistry^41^,^42^. The change in solvation free energy of the proton between bulk solution and adsorbed at the mineral surface depends primarily on the mineral’s dielectric constant and provides the interfacial solvation energy term. The crystal chemistry term accounts for electrostatic interactions and H-bonding at the mineral surface. This term includes parameters such as chemical composition, stoichiometry, coordination geometry, Pauling bond strength and average bond-lengths. Thus, vesicle formation rates are ultimately controlled by the fundamental chemical, structural and physical properties of the minerals.

Initial vesicle formation-rates in both environments also depended on the specific surface area of the minerals available for interaction with the vesicles (Fig. 2). For minerals with specific surface areas ~0.5–35 m^2^.g^−1^, the initial rates increased by ~100–1000 fold as mineral surface charge became more positive (Fig. 2 and Extended Data Figures 3 and 4). The nanoparticulate oxides have significantly larger specific surface areas (>50 m^2^.g^−1^) and follow the same trend with IEP as the larger particles, but the rate enhancement appeared to be about an order of magnitude smaller, which is non-intuitive. This effect can be explained by the knowledge that nanoparticles tend to flocculate and form larger clusters than suggested by the size of the discrete, individual grains (Fig. 1 and Extended Data Figure 1). This affects the mineral surface area available for lipid interaction. In detail, the N_2_ gas molecules that are used to determine the specific surface area are ~1 Å in size, so they can sample the surface of both the mineral clusters as well as the surface of the individual discrete particles within such clusters. In contrast, the lipid molecules are ~1–2 nm in size and cannot completely sample the surface area of the particles within the cluster. Thus, the specific area determined by N_2_ gas adsorption isotherm ends up being an overestimate of the surface area actually available for interaction with the much larger lipid molecules. This is a well-known phenomenon in mineral-water interface geochemistry and colloid chemistry, and attempts to estimate effective surface area have largely been unsuccessful^43^,^44^.

Siderite appeared to be an exception to the size-dependence trend (Fig. 2A). Our sample is a natural mineral, so Mg^2+^ and Ca^2+^ were partially substituted into the Fe^2+^ sites during the original crystal growth. In solution, ions were leached out (Extended Data Figure 6A), Fe^2+^ was oxidized to Fe^3+^ and was precipitated as nanoparticulate goethite on siderite, indicated by a change in solution color to reddish-brown. Goethite formed at room temperature is nanoparticulate and would aggregate, thus resulting in lower vesicle formation-rates. To test this interpretation, we synthesized goethite in solution (Methods). Nanoparticulate size and high surface area were obtained (Table 1 and Extended Data Figure 1) and particle aggregation was observed (Fig. 1). The initial vesicle formation rates were similar to those for siderite consistent with our prediction (Fig. 2A). The initial rates of siderite and goethite were also similar to each other for vesicle formation by lipid thin film rehydration (Fig. 2B).

In summary, minerals positively influenced the initial formation rates of vesicles (protocell membrane self-assembly) composed on negatively-charged and neutrally-charged SCAs at concentrations above the lipid CVCs.

### Critical Vesicle Concentration

**Figure 4:**
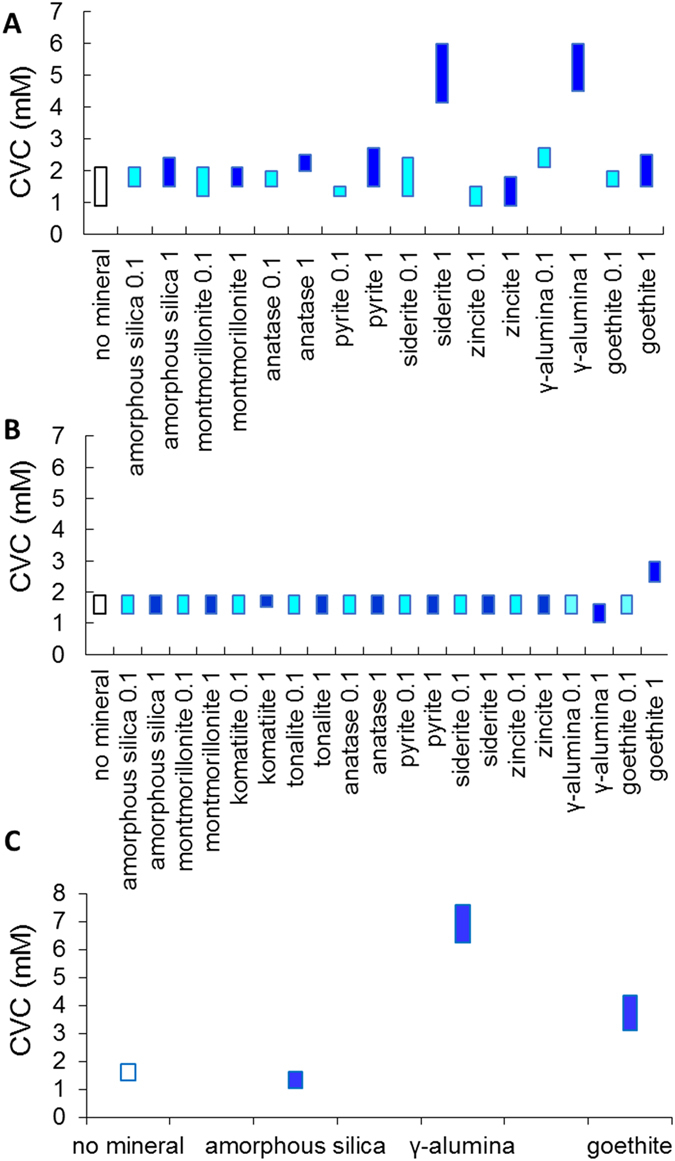
Summary of mineral effects on CVC of DA/DOH (2:1). CVC as determined by (**A**) DLS and (**B**,**C**) fluorescence, at pH 8.1 and particle loadings of 0.1 and 1.0 mg.mL^−1^. Little or no effect was observed except in the case of siderite, γ-alumina and goethite at high loading; (**C**) effect of settling of positively-charged minerals compared to negatively-charged silica which provides a positive control. See Methods and Extended Data Figures 7 and 8 for details.

Mineral effects on CVC of DA/DOH were determined by two different methods, dynamic light scattering (DLS) and a fluorescence assay (Fig. 4). In the DLS assay, mineral particles were added to pre-formed vesicles and vesicle formation was marked by a sharp increase in scattering intensity (Methods; Extended Data Figure 6). In the second assay, and a fluorescent dye was encapsulated into the vesicle membrane during lipid thin film rehydration (Methods; Extended Data Figures 7). Both assays have been used previously as a measure of vesicle formation from SCAs in the absence of minerals^13^,^34^, ^35^, ^36^, ^37^. In the present study, a CVC of ~1–2.5 mM was obtained in both assays without any minerals present consistent with previous work^6^. Addition of most minerals did not affect the CVC (Fig. 4A,B), suggesting that the fraction of lipid removed from solution by adsorption is small. Only goethite, siderite (altered surface layer of goethite) and γ-Al_2_O_3_ at higher mineral loading resulted in a 2- to 3- fold increase in CVC. These are positively-charged minerals, so more negatively-charged lipid is expected to associate; subsequent, aggregation and settling of such particles at higher loadings should remove the associated lipid from solution. More lipid would then be required to form vesicles, thus apparently increasing the CVC. Conversely, a smaller amount of lipid would be associated with negatively-charged surfaces and would not affect the CVC even upon aggregation and settling. This interpretation is supported by the determination that negatively-charged silica has no effect on CVC even at high loading (Fig. 4C and Extended Data Figure 8). The formation of larger lipid-mineral aggregates in the case of γ-Al_2_O_3_ compared to amorphous silica was also observed by eye in micro- test tubes and by phase contrast microscopy (images not shown).

Additionally, in the case of siderite, a total cation concentration of ~1.7 mM at 1.0 mg.mL^−1^ was leached into solution (Extended Data Figure 6A). Divalent cations are known to bind strongly to fatty acid head groups and cause lipid aggregation and settling, thus removing the lipid from solution^25^ and increasing CVC. This phenomenon likely also occurred in the presence of divalent cations leached from siderite (Fig. 4a). This interpretation was supported by the results of phase contrast microscopy of DA in the presence of added dissolved Mg^2+^ without any minerals present (Extended Data Figure 6B; Methods). Abundant vesicles were observed in the absence of Mg^2+^; a few aggregates appeared at 0.5 mM MgCl_2_, although vesicles were still predominant; and substantially more aggregation was seen by 1.5 mM MgCl_2_. In summary, mineral solubility and particle settling at high loading of positively-charged nanoparticulate minerals influenced CVC. Thus, some minerals at high particle loadings may have had an unfavorable thermodynamic effect on the ability for cell membranes to form in conditions where lipid concentrations were close to the CVC.

### Membrane Permeability

Membrane permeability was determined by monitoring calcein leakage from vesicles (Extended Data Figure 9; Methods). Leakage depended on the type of lipid (Extended Data Figure 9C). OA and POPC were almost impermeable to calcein over the time period of the experiment, in contrast to ~40% leakage from the DA/DOH vesicles. These results confirm previous findings that longer tail lipids with a C=C double-bond (OA) and phospholipids with two fatty tails (POPC) are more impermeable than vesicles composed of short chain aliphatic tails^7^,^45^. Also, calcein leakage varied inversely as the total lipid concentration from 25 to 7 mM (Extended Data Figure 9D).

To study the effect of minerals, we attempted to maximize the lipid-mineral interactions by using lower a total lipid concentration of 7 mM. Vesicles exhibited a biphasic leakage behavior with a breakpoint at ~2 h in the leakage curve (Extended Data Figure 9E). The first phase is related to calcein leakage. After 2 h, we observed some evaporation of solution from the small sample volume (Methods), which probably resulted in vesicle rupture, as reflected in the breakpoint of the leakage curve. None of the minerals (0.1 mg.mL^−1^) had any appreciable effect on membrane permeability for the first 2 h, probably reflecting a large excess of lipid over minerals even at the lower lipid concentration (7 mM). The experiment was then conducted at higher particle loadings of 0.25 and 0.5 mg.ml^−1^, but no significant effect of the minerals on the leakage of calcein was observed (data not shown). However, it was noticed that the second phase (vesicle rupture) was often delayed, although without an identifiable mineral-specific trend. This observation was interpreted as a process in which a small fraction of vesicles adsorbed intact on the mineral surface, which delayed their rupture. Finally, it is worthwhile to mention that minerals also had no effect on the calcein leakage from phospholipid (POPC) and oleic acid (OA) vesicles or from of DA/DOH (2:1, total lipid = 25 mM) vesicles (data not shown). These results indicate that once vesicles have formed, contact with mineral surface does not lead to their rupture at least at high lipid/mineral ratios. Thus, protocell membranes would have survived in contact with mineral surfaces.

### Mechanism for Acceleration of Vesicle Formation in the Presence of Minerals

The catalytic effect of vesicle surfaces in micelle-to-vesicle transition leading to vesicle growth has been noted previously in the absence or presence of mineral surfaces. This has been termed the “matrix effect” and “intermediate lipid structures” have been invoked but the detailed mechanism for the catalytic effect is still not clear^13^,^14^,^34^,^35^,^36^,^38^,^39^. In the presence of minerals, micelle-to-vesicle transition rates were faster and even teflon, a hydrophobic and lyophobic material, promoted the rate when coated with amphiphile^14^. Vesicle formation appeared proximal to, but not in direct contact with, the mineral surface. It was proposed that an increase in local concentration of amphiphiles near the mineral surface leads to larger lipid aggregates, which are then released to form vesicles. Thus, the mineral surface seemed to provide a “matrix” similar to vesicle surfaces in the absence of minerals. However, no dependence on specific mineral properties was identified and the physical interactions responsible for the proximity-effect was unexplained in previous work.

The results in the present study confirm the micelle-to-vesicle transition rate in the presence of minerals. The present work goes further in demonstrating a similar effect for *de novo* vesicle formation by lipid thin film rehydration and also shows that minerals can influence apparent CVC. Significantly, correlations were identified between the initial vesicle formation rates and the IEP of the mineral as well as the amount of lipid adsorbed and the mineral surface charge. DA and DA/DOH adsorb on both negatively-charged and positively-charged mineral surfaces, because adsorption is controlled by a combination of van der Waals, H-bonding and electrostatic forces. Similar correlations have been identified previously for phospholipid membrane stability at oxide surfaces and for cytotoxicity of oxide nanoparticles on bacterial cell surfaces^19^,^20^,^21^,^22^,^23^. In detail, by using a combination of adsorption isotherms, fluorescent dye (calcein) leakage rate experiments, atomic force microscopy, neutron reflectivity and transmission electron microscopy, it was shown that dipalmitoylphosphocholine (DPPC) and ditridecanoylphosphocholine (DTPC) adsorb by forming incomplete multi-layer islands on the surfaces of quartz, rutile, mica and corundum (α-Al_2_O_3_)^19^,^20^,^21^,^22^. Furthermore, a modified Deraguin-Landau-Verwey-Overbeek (DLVO) theory was developed and combined with adsorption isotherms to explain how the electrostatic effect of the mineral’s surface charge is effective even up to 12 or 18 nm distance from the surface through 2 or 3 stacked bilayers of lipid on silica or alumina, respectively. Specifically, multiple (though incomplete) layers of lipid can adsorb because of van der Waals interactions between adjacent layers. The presence of these lipid layers results in exclusion of solvent and counterion from the proximity of the mineral surface. Thus, the electric double layer is effectively extended away from the surface^21^.

We propose that similar islands of DA or DA/DOH form on the minerals in the present study and they can be piled up to several nanometers away from the surface (Fig. 3). These islands catalyze the formation of vesicles. Complete coating of the mineral surface with lipids is not required for the catalytic effect but initial adsorption of some lipid is needed. This scenario is consistent with the ideas proposed by Hanczyc *et al*.^14^. Our study goes further in establishing the SARs, and in providing a theoretical basis for the dependence on surface charge and for why the catalytic effect can apparently occur without direct contact with the mineral surface^21^.

## Conclusions

We have shown here that the effects of minerals on the self-assembly of SCA vesicles are related to the IEPs and available reactive surface areas of the minerals. Adsorption is a key necessary step for minerals to affect vesicle formation rates and CVC. Even a small amount of adsorption is sufficient to catalyze vesicle self-assembly, but a large amount of lipid adsorption and removal from suspension is required to influence the thermodynamics of vesicle self-assembly (CVC) Membrane, membrane integrity of pre-formed vesicles is hardly affected by minerals. Thus, minerals could have influenced the thermodynamics and kinetics of initial self-assembly of protocell membranes but, once formed, the protocells could have survived in contact with rock surfaces. Finally, we have shown here that the reactive surface area available for reaction is crucial, rather than the B.E.T. specific area determined by N_2_ gas adsorption, in the catalysis phenomenon.

Mineral IEPs ultimately depend on their fundamental crystal structure, ion coordination in the crystal, chemical composition and bulk dielectric constant. We used minerals and rocks to represent early Earth environments, encompass a wide range of physicochemical properties and represent low- and high-energy environments. Therefore, we believe that the structure-activity relationships identified are robust and should apply to other minerals not included here, with broader implications for other biogeochemical processes as well as biomedical, industrial and technological applications. The results of the present study may help predict protocell membrane formation on Mars and Moon, and other solid worlds such as Io, Ganymede, Enceladus and even exo-solar planets once their mineralogy becomes known.

## Methods

### Mineral characterization

The mineral and rock samples used were either synthetic swap positions of synthetic and natural. Commercially purchased samples were synthetic amorphous silica (SiO_2_, Aerosil^®^ 300, Evonik, Essen, Germany); synthetic γ-alumina (γ-Al_2_O_3_, 99%), pyrite (FeS_2_, 99.8%), rutile (α-TiO_2_, 99.99%), anatase (β-TiO_2_, ≥99%), and zincite (ZnO, 99.999%) all from Sigma Aldrich (Saint Louis, MO, USA); natural quartz (α-SiO_2_, Minusil^®^ 5, U.S. Silica, Pacific, MO, USA); natural siderite (FeCO_3_, Nova Scotia; purchased from Ward’s Science, Rochester, NY, USA) and natural montmorillonite volclay^®^(Wyoming SPV 200, American Colloid Company, Troy, IN, USA). Volclay is a bentonite from Wyoming composed primarily of montmorillonite and minor amounts of muscovite, quartz and feldspar. Specimens of komatiite (~ 20 weight % MgO; Spinifex Hill, Barberton Formation, S. Africa, age <~3 Ga) and tonalite (Biron Dam, Wilson Rapids, WI, age ~1.8 Ga) were kindly donated by Dr. Phillip Brown, Department of Geoscience, University of Wisconsin-Madison. Siderite, tonalite, and komatiite were received as whole-rock samples, so it was necessary to crush them using either a stainless steel ball-mill or agate mortar and pestle until powders of less than 325 mesh (72 μm) were obtained. Smaller particle sizes could not be achieved.

All minerals were characterized by powder X-Ray Diffractometry (Ultima IV Rigaku, DE, USA) to confirm their crystal structures. The specific surface area of the minerals used was determined by multipoint N_2_ gas adsorption (Micromeritics, GA, USA and Quantachrome, FL, USA) using the B.E.T. formalism (Table 1).

The minerals and rocks used in the present study were further characterized by Transmission Electron Microscopy (TEM, JSM-1230, ~120 kV, JEOL, MA, USA). 1–2 μL of 0.1 mg.mL^−1^ mineral suspension were placed on 300-mesh copper grids coated with formvar/carbon film and air dried. They were also characterized by phase contrast microscopy (Olympus IX51) equipped with sCMOS camera (QImaging, optiMOS) and cellSens Dimension 1.7 software, in both HEPES (200 mM, pH 7.1) and bicine (200 mM, pH 8.1) buffers. The visualization was performed using an Olympus 100x oil objective.

The IEPs of minerals were determined by measuring the zeta potential using a Zetasizer (NanoSeries ZS, Malvern Instruments, UK) (Table 1). Samples were prepared at 1 mg.mL^−1^ mineral suspensions in 18 M-cm ultrapure water and pH was adjusted from 2 to 10 by adding HCl or NaOH.

The concentration of Ca^2+^, Mg^2+^, and total Fe (Fe^2+^ + Fe^3+^), which may leach out from the minerals when immersed in buffer, was determined by inductively coupled plasma-optical emission spectrometry (ICP-OES, 710 ICP-emission spectrometer, Agilent Technologies, Santa Clara, CA, USA). A stock solution (10 mg.mL^−1^) of siderite, montmorillonite, tonalite, and komatiite were pre-equilibrated (3 days) in bicine buffer (200 mM, pH 8.1). 0.1 and 1 mg.mL^−1^ dilutions were made in buffer and further equilibrated on an end-over-end rotator at 40 rpm overnight. Afterwards, samples were centrifuged at 4000 rpm for 10 minutes, the supernatant was then filtered (0.2 μm) and analyzed using calibrated standards (High Purity Standards, Charleston, SC, USA).

### Goethite synthesis

Goethite was prepared using a slightly modified version of the method described by van Geen and coworkers^46^. Briefly, 45 g of ferric nitrate (Fe(NO_3_)_3_•9H_2_O) was dissolved in 830 mL of ultrapure water (18 M-cm, CO_2_ degassed by boiling) in a glove bag (Coy Laboratory Products Inc., Grass Lake, MI, USA) purged with N_2_ gas. Then, 90 mL of 5 N NaOH (99.99% pure) was added to the ferric nitrate solution and a red precipitate was immediately formed. The suspension was stirred for 2 hours. An aliquot of 50 mL was kept at room temperature and the rest was transferred to a 2 L polypropylene bottle. This entire procedure was conducted in the glove bag. The bottle was capped and placed in a furnace (Thermo Lindberg/Blue M™ Furnace; Thermo Fisher Scientific, Waltham, MA, USA) at 60 ± 4 °C for 24 hours. The suspension was then placed in trace metal-free dialysis tubing (Spectra/Por^^®^^ 6, Spectrum Labs, Rancho Dominguez, CA, USA) and dialyzed against ultrapure water. The water was changed once or twice a day and the conductivity and the pH of the spent water were measured. This process was continued for two changes past the point where the conductivity of the solution in contact with the goethite for one day was 1–2 μS.cm^−1^, or where the solution pH remained constant at ~7.5–8. The goethite suspension was centrifuged at 2000 rpm for 5 min, the supernatant was discarded and the remaining slurry was freeze-dried (FreeZone 4.5 Liter Console Freeze Dry System, Labconco, Kansas City, MO, USA). The preparation yielded ~15 g of goethite, whose structure was confirmed by XRD. The specific surface area of the dried goethite powder was determined by performing a multipoint N_2_ gas adsorption-desorption isotherm at 77 K (Micromeritics Tristar II 3020 analyzer) and fitting to a B.E.T. model, which yielded a value of 63 ± 2 m^2^.g^−1^.

### Microscopy of Minerals and Lipids

#### Cryo-TEM

Suspensions of minerals/vesicles were prepared by mixing DA micelles (75 mM) with the studied mineral (5 mg.mL^−1^) at pH 7.8. Note that this pH was an exception (pH was 7.1 for the rest of the DA systems) for this experiment. This was because a coexistence of the vesicular and micellar phases of DA is expected at pH 7.8, so, it was hoped that the micelle/vesicle transition could be captured. The buffer concentration was also lowered to 50 mM, to minimize the possibility of buffer crystals in the sample, which had been observed in previous trials with 200 mM buffer. Vitrified thin specimens were prepared using a plunge freezer (FEI Mark IV Vitrobot, Hillsboro, OR, USA) set at room temperature and ~95% humidity. About 2.5 μL of the solution was applied to a TEM grid coated with lacey carbon film. After blotting using two filter papers, the grid was plunge-frozen in liquid ethane^47^. The vitrified specimen was mounted onto a Gatan 626.DH cryo-holder and transferred into a FEI Tecnai F20 TEM equipped with a Gatan twin blade retractable anticontaminator. The cryo-TEM was carried out at ~−174 °C.

#### Phase-Contrast Microscopy

The co-existence of vesicles and minerals was confirmed using phase-contrast microscopy. For DA (200 mM) system, a solution of micelles prepared at pH 12 (200 mM NaOH) was mixed with mineral suspension prepared in HEPES buffer (200 mM, pH 7.1) to obtain a final lipid concentration of 30 mM, final pH 7.1 ± 0.1 final particle loading of 1 mg.mL^−1^. The pH measured for all samples was of 7.1 ± 0.2. For the DA/DOH system (2:1), vesicles prepared by sonication and vortexing in bicine buffer (200 mM, pH 8.1) were mixed with minerals prepared in the same buffer, to obtain a final lipid concentration of 10 mM, final pH 8.1 and a final particle loading of 1 mg.mL^−1^. For each system, 5 μL of the lipid/mineral mixture was placed between a glass slide and a cover slip, and observed in phase contrast mode using a light microscope (Olympus IX51).

### Kinetics of DA vesicle formation by micelle-to-vesicle phase transition analyzed by UV/Vis spectroscopy

The procedure was adopted from a previous report^14^. Mineral particles suspended in water (10 mg.mL^−1^) were mixed with HEPES pH 7.1 (1 M) in a semi-micro disposable cuvette (BrandTech^^®^^ Scientific Inc., Essex, CT, USA). The reaction was started by adding DA micelles (200 mM) at pH 12 (200 mM NaOH), to reach a final concentration of 30 mM DA at pH 7.1 (HEPES, 200 mM) and a final particle loading ranging from 0.01 to 2 mg.mL^−1^ in 1 mL total volume. The experiment was conducted at 25 °C. The turbidity, hence, absorbance of the solution increases as micelles transform to vesicles (Extended Data Figure 2). The turbidity was measured at 400 nm every second for 12 minutes (Extended Data Figure 3). The baseline with mineral alone in buffer (no DA present) was subtracted from the corresponding spectrum to minimize interference to the signal from mineral settling. To determine the initial rate of vesicle formation (*V*_*i*_), the constant rate at each particle loading was determined up to the first 2 minutes of the reaction using the equation: ln *A*_*t*_/*A*_*0*_ = – *V*_*i*_.*t*, where *A*_*t*_ is the absorbance at time *t, A*_*0*_ is the absorbance at time zero and *V*_*i*_ is the apparent initial rate. For each mineral, the initial rates were plotted as a function of the total mineral surface area (Extended Data Figure 3). The slope of the obtained linear graph reflected the initial rates per unit specific surface area (*A.s*^*−1*^*.m*^*−2*^). This parameter is an intrinsic constant with respect to each mineral, and allows for comparison between different minerals (Extended Data Figure 3). All experiments were conducted in triplicate and repeated at least twice. The error bars of the slopes were determined by using the function LINEST of Excel 2013.

### Kinetics of DA/DOH (2:1) vesicle formation by rehydration of dehydrated lipid thin films analyzed by fluorescence spectroscopy

The procedure has been modified from previous reports^36^,^48^ and references therein. Briefly, vesicles were formed by rehydrating a thin film of DA/DOH (2:1) with mineral suspension to a final pH of 8.1 ± 0.2. The experiment was conducted at 25 °C. Kinetics were determined by measuring the change in fluorescence intensity of naphthopyrene (NP), a membrane-soluble dye, over 3 hours. In detail, stock solutions of 50 mM of DA/DOH and 0.01 mM of NP were dissolved in chloroform. Aliquots of the stock solutions were dispatched into a 96-well glass microplate to achieve a final concentration of 10 mM lipid and 1 μM NP. Chloroform was removed by 3–4 hours of drying under vacuum, which resulted in a lipid thin film. Subsequently, the film was rehydrated with 250 μL of mineral suspensions (0.01 to 1 mg.mL^−1^) at pH 8.5 (bicine, 200 mM). Note that this initial buffer pH was chosen to be higher than 8.1, since it was noticed that the pH systematically drops to ~8.1 after dissolving all the lipid. Kinetics of vesicle formation were recorded using a plate reader (Synergy H1 microplate reader, Bio-Tek Instruments, Inc., Winooski, VT, USA) at 292/465 nm (excitation/emission) at regular intervals of 30 seconds for 3 hours. During the measurement, the plate was kept on continuous orbital shake at 425 cpm. The initial period (0 to 900 s) was used to determine the initial rate, *V*_*i*_, of vesicle formation. Experiments were done at least in duplicate and each experiment was repeated at least two times. The initial rates and initial rates.m^−2^ were calculated similarly to those obtained in the UV-Vis experiments (see previous section for details).

### CVC determination using dynamic light scattering

The procedure was modified from previous reports^43^, ^44^, ^45^. A stock solution of 300 mM DA/DOH (2:1) was by rehydrated a lipid thin film with bicine buffer (200 mM) to a final pH of 8.1. This stock of pre-formed vesicles was diluted to 30 mM with bicine pH 8.1. All lipid solutions were stored on a stir plate (60 rpm), and were allowed to equilibrate for a minimum of 2 hours prior to their use. The experiments were conducted at 25 °C. The CVC of DA/DOH with and without the presence of mineral suspensions at 0.1 and 1 mg.mL^−1^ was determined using DLS. For each experiment, twenty-six aliquots of 1.5 mL of DA/DOH (0–18 mM) were prepared at pH 8.1 (bicine, 200 mM). In experiments with minerals, mineral-buffer suspensions (10 mg.mL^−1^) were added to the aliquots to achieve final loadings of 0.1 mg.mL^−1^ or 1 mg.mL^−1^. These solutions were allowed to equilibrate at 40 rpm for a minimum of 12 hours.

Dynamic Light Scattering measurements were conducted at 25 °C with 173° back-scattering using a ZetaSizer NanoSeries (Malvern Instruments, London, UK) in disposable semi-micro cuvette (BrandTech 759150, Brand GmbH, Wertheim, Germany). Following previous literature^49^,^50^,^51^, the CVC was determined as the concentration at which a substantial increase in the amount of scattered light was observed relative to a baseline established by low DA/DOH concentrations. Although the amount of interference from mineral particles was minimized by gravitational settling, a significant variation of light scattering was observed, nevertheless, at low DA/DOH concentrations. The resulting variable baseline prevented us from assigning a single DA/DOH concentration as the CVC. Instead, a range of DA/DOH concentrations was identified as the CVC. All experiments were conducted at least three times.

### CVC determination using fluorescence spectroscopy

In addition to DLS, the CVC of a DA/DOH (2:1) mixture at 25 °C was also determined by measuring the NP fluorescence intensity. Stock solutions of 25 mM lipid and 0.01 mM NP were prepared in chloroform. Aliquots of lipid and NP stock solutions were dispatched to a 96-well glass microplate (Zinsser NA, CA, USA) to achieve various (0–10 mM) final concentrations of lipid and a 1 μM final concentration of NP. A lipid thin-film was formed after 3–4 h of vacuum-drying of the well plate containing lipid. Subsequently, the film was rehydrated with 250 μL of mineral suspension in bicine buffer (200 mM, pH 8.5) to obtain a final pH of 8.1 ± 0.2. A 96-well microplate contained one “no mineral” control and triplicate samples of a specific mineral. Fluorescence was recorded on a plate reader (Synergy H1 microplate reader, Bio-Tek Instruments, Inc., Winooski, VT, USA) using single-point measurements at 292/465 nm (excitation/emission). During the measurement, the plate was kept on continuous orbital shake at 425 cpm. The data collected after 1 hour were used for the determination of the CVC. All experiments were conducted in triplicate and repeated at least three times.

To study the potential effect of mineral-lipid aggregating and settling on the CVC of DA/DOH, another set of experiments was performed using the same assay. A series of lipid thin films with concentration ranging from 0–15 mM with 1 μM NP were rehydrated with 1 mL of γ-Al_2_O_3_ or goethite or amorphous silica suspensions (0.1 and 1 mg.mL^−1^) (bicine, 200 mM, pH 8.5) to a final pH of 8.1 ± 0.2. These suspensions were allowed to equilibrate at 40 rpm for 1 hour with end-over-end rotation (SB3, Stuart rotator). Subsequently, 250 μL of each suspension was taken and the fluorescence was immediately measured. The remaining mineral-vesicle suspension in the eppendorf tubes was left undisturbed for 2 hours to allow settling of the mineral. Subsequently, fluorescence was recorded for each supernatant collected. All experiments were conducted in triplicate and repeated at least three times. CVCs measured by fluorescence for the lipid alone (“no-mineral” control) and in the presence of minerals are shown in Fig. 4 and Extended Data Figure 8.

### Adsorption of DA and DA/DOH on silica and alumina columns

Three different total lipid concentrations (10, 25 and 50 μmol) were incubated in triplicate with 0.2 g of either alumina gel (ThermoFisher, Waltham, MA, USA) or silica (Sorbent Technologies™, Norcross, GA, USA) gel, for 24 hours. In detail, for the DA system, 200 mM micelles (in 200 mM NaOH, pH 12) were mixed with the mineral suspension prepared in HEPES buffer (200 mM, pH 7.1), to obtain the final lipid concentrations mentioned above. For the DA/DOH system, 100 mM DA/DOH vesicles prepared by sonicating and vortexing in bicine buffer (200 mM, pH 8.1) were mixed with the mineral suspension prepared in bicine buffer (200 mM, pH 8.1). The pH of the samples after 24 h incubation was: DA/silica, pH = 7.1 ± 0.1; DA/alumina, pH = 7.3 ± 0.2; DA/DOH/silica, pH = 8.1 ± 0.1; DA/DOH/alumina, pH = 8.4 ± 0.2. After incubation, 5 mL disposable columns were packed with the lipid/mineral mixture and the eluents were collected. Columns were washed with 1 equivalent volume of the corresponding buffer and the volume of the total eluents was measured. Lipid concentrations in the eluents were determined by HPLC (Nexera 2, Shimadzu^^®^^, Columbia, MD, USA) as compared, respectively, to DA and DA/DOH standard curves. The lipid separation was done on a Kinetex 2.6 μm Biphenyl 100 A column, 100 × 4.6 mm (Phenomenex^®^, Torrance, CA, USA) using a mobile phase of MeCN/H_2_O (60:40) at a flow rate of 1 mL.min^−1^. The mobile phase was acidified with 0.1% TFA to stabilize the DA in its associated form. The UV detection wavelength was set at 202 nm using UV–Vis photodiode array detector (SPD-M30A, Shimadzu^®^). Separation was carried out at 40 °C. Error bars represent standard deviation of triplicate samples and the entire experiment was repeated twice.

### Phase Contrast Microscopy for Dissolved Cation Effects

DA vesicles without and with dissolved MgCl_2_ present in solution were imaged with phase contrast light microscopy (Olympus IX51) equipped with sCMOS camera (QImaging, optiMOS) and cellSens Dimension 1.7 software. DA (100 mM) vesicles were prepared at pH 7.1 (HEPES, 200 mM). A variable amount of a 50 mM MgCl_2_ solution was added to the vesicle solutions to obtain a final MgCl_2_ concentration ranging from 0 to 15 mM and DA concentration of 50 mM. Within five minutes of adding MgCl_2_ to DA vesicles, the mixture was put on a glass slide and viewed with a 40x or 100x oil objective lens (Extended Data Figure 6B).

### Calcein leakage assay

Briefly, this assay is based on the encapsulation of calcein, a fluorescent dye, at its self-quenching concentration^8^,^52^. Vesicles are then separated from the bulk free dye, and the leakage of calcein from the vesicles is monitored over time by the increase in fluorescence resulting from the de-quenching of calcein as it leaks out from the vesicles.

A DA/DOH (2:1) lipid film was rehydrated with a solution of 20 mM calcein at in bicine buffer (200 mM, pH 8) to achieve a final lipid concentration of 300 mM. After brief sonication, the pH was re-adjusted to 8 ± 0.1 with NaOH and the sample was allowed to equilibrate overnight. Vesicles were extruded through a 200 nm polycarbonate membrane using a mini-extruder (Avanti^®^ Polar Lipids, Alabaster, AL, USA) to form homogenous, monodisperse and single-bilayer vesicles (Extended Data Figure 9A). Low-pressure size-exclusion chromatography was used to separate the calcein dye-encapsulating vesicles from the un-encapsulated, free dye. An empty glass Econo-column^®^ (Bio-Rad) of dimensions 25 cm × 1 cm was filled with Sephadex G-50 medium beads (Sigma Aldrich). The mobile phase was 200 mM bicine buffer at pH 8. The flow rate was maintained at 1.5 mL.min^−1^ (Econo gradient pump, Bio-Rad) and the injection volume was of 300 μL. Fractions were collected (FC204, Gilson) in a 96-micro-wellplate (6 drops/well) and the fluorescence was measured in a plate reader (Synergy H1, BioTek) at Ex/Em of 492/530 (Extended Data Figure 9B). The DA/DOH purification achieved was never better than that shown in Extended Data Figure 9B, no matter what was the length or the width of the column. In other words, DA/DOH were too leaky and the calcein leaked out of the vesicles as soon as the vesicles were separated on the column, consistent with a previous report^53^.

The fractions containing vesicles were pooled and purified vesicles were mixed with each mineral in a 96-well plate. The volume of the mixture was 100 μL and the final particle loading varied from 0.1 to 0.5 mg.mL^−1^, in the presence and absence of Triton^TM^-100X (0.1%), each condition in triplicate. The addition of Triton ruptures the vesicles immediately and, thus, the self-quenched encapsulated calcein leaks out and unquenchs, leading to a maximum fluorescence intensity, which is considered as 100% leakage. Kinetics of calcein leakage were recorded in a plate reader every 5 minutes for 16 hours and the percentage of encapsulation was calculated according to the equation 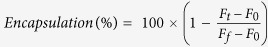, where *F*_*t*_ is the fluorescence at time t, *F*_*0*_ is the fluorescence at time zero and *F*_*f*_ is the final fluorescence after addition of Triton (rupture of vesicle membrane). It is worth it noting that during the measurements, a decrease of the intensity of the triton-controls was noticed after about 6–9 hours of incubation, indicating a requenching of calcein. This was due to the slow evaporation of the sample during the measurements. This process also controlled the rupture of the vesicles, which ultimately migrate to the wall of the well and break (small volume and no shaking). The rupture was indicated by the breakpoint of the graphs in Extended Data Figure 9E. However, the presence of minerals seemed to constantly delay the rupture of the vesicles.

## Additional Information

**How to cite this article:** Sahai, N. *et al*. Mineral Surface Chemistry and Nanoparticle-aggregation Control Membrane Self-Assembly. *Sci. Rep.* **7**, 43418; doi: 10.1038/srep43418 (2017).

**Publisher's note:** Springer Nature remains neutral with regard to jurisdictional claims in published maps and institutional affiliations.

## Supplementary Material

Supplementary Information

## Figures and Tables

**Table 1:** Mineral characterization.

Mineral/rock	Stoichiometry and crystal phase	Isoelectric point (IEP)#	B.E.T. specific surface area (m^2^.g^−1^)¥	Individual particle size, cluster size by conventional TEM (nm)§
silica (Aerosil-300)	amorphous SiO_2_	1.5–2.5	288 ± 5	15, 50–500
quartz (Minusil-5)	α-SiO_2_	2–3	6 ± 0.5	>100
montmorillonite	Na_0.2_Ca_0.1_Al_2_Si_4_	1.5–2.5	33 ± 1	>200
(Volclay SP-200)*	O_10_(OH)_2_(H_2_O)_10_			
komatiite (rock)	oceanic crust	1.5–2.5	3 ± 0.5	>300
tonalite (rock)	continental crust	3–4	6 ± 0.5	>300
rutile	α-TiO_2_	4–5	0.7 ± 0.2	>300
anatase	β-TiO_2_	5.4–6	54 ± 2	15, 50–500
pyrite	FeS_2_	6.3–7.3^54^	0.5 ± 0.1	>1000
siderite	FeCO_3_	7.2–7.6	9 ± 0.5≠	>1000
zincite	ZnO	8.3–8.7	3 ± 0.3	>300
γ-alumina	γ-Al_2_O_3_	8.3–9.3	119 ± 3	15 nm wide, 100 nm long; 50–500
goethite	α-FeO(OH)	8.7–9.3	63 ± 2	15 nm wide, 100 nm long; >50–500

See Methods for details.

^#^Determined by measuring ζ-potential of minerals at different pH in ultrapure water.

^¥^Measured using nitrogen gas adsorption-desorption isotherm at 77 K.

^≠^Measured after equilibration with buffer for 24–48 h.

^*^Commercial bentonite from Wyoming and is predominantly composed of montmorillonite.

^§^Estimated from conventional TEM images (Extended Data Figure 1).
