# Supplementary material for: Mineral Surface Chemistry and Nanoparticle-aggregation Control Membrane Self-Assembly
Source: Sci Rep. 2017 Mar 7;7:43418. doi: 10.1038/srep43418 (PMC5339912; doi:10.1038/srep43418)
Supplement: Supplementary Information [file srep43418-s1.pdf]

Scientific Reports

Revised: December 2, 2016

Supplementary information for

Mineral Surface Chemistry and Nanoparticle-aggregation Control Membrane Self-Assembly

Nita Sahai [1, 2, 3, *], Hussein Kaddour[1], Punam Dalai[1], Ziqiu Wang[1], Garrett Bass[1], and Min Gao[4]

[1]Department of Polymer Science, University of Akron, Akron, OH 44325, USA.

[2]Department of Geology, University of Akron, Akron, OH 44325, USA.

[3]Integrated Bioscience Program, University of Akron, Akron, OH 44325, USA.

[4]Liquid Crystal Institute, Kent State University, Kent, OH 44240.

[*]corresponding author: Email address: sahai@uakron.edu, Tel.: +1 330-972-5795,

170 University Avenue, University of Akron, Akron, OH 44325-3909, USA

<u>This file contains:</u> Extended Data figures 1 to 9

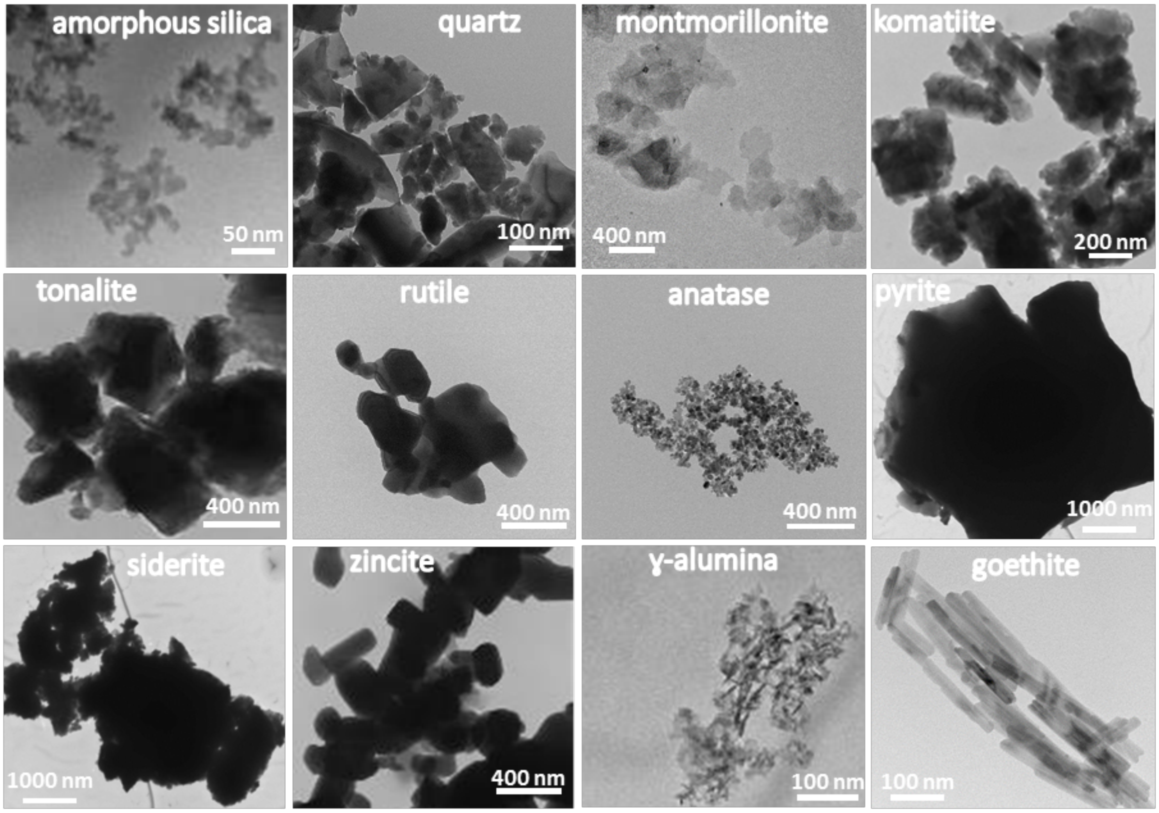

Extended Data Figure 1. TEM bright field images of rocks and minerals used in this study. See Methods for details.

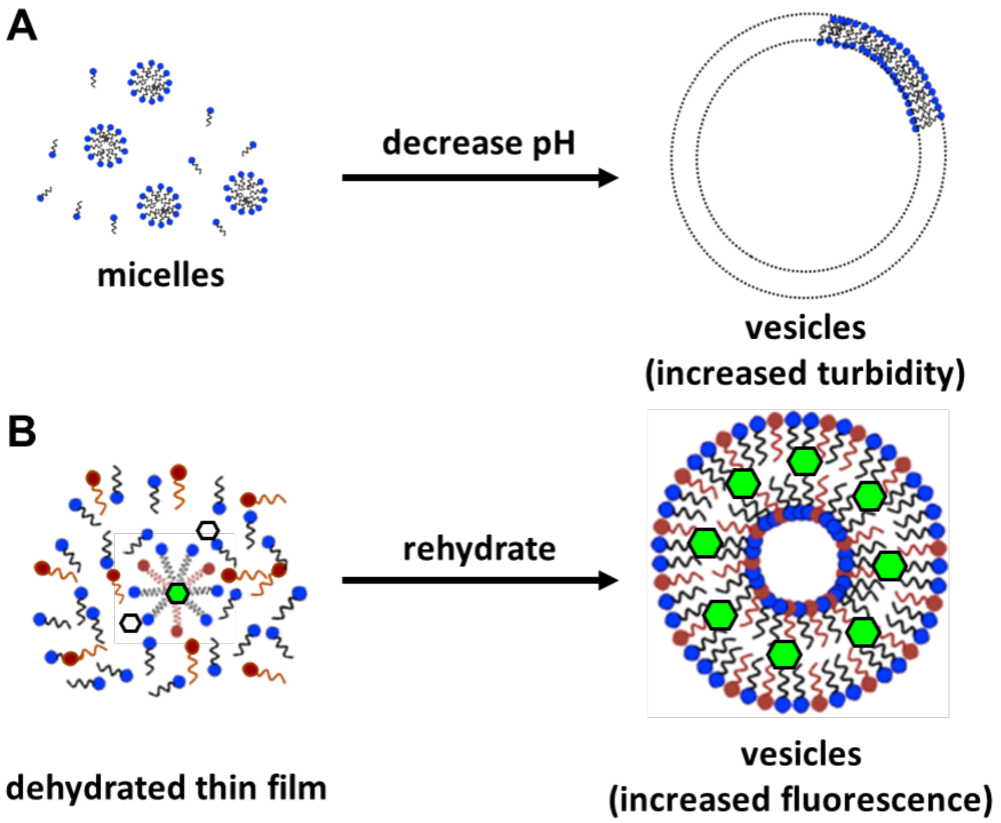

Extended Data Figure 2. Schematic illustrating the principles for determining the vesicle formation. (A) micelle-to-vesicle formation by reducing pH from 12 to 7. Vesicle formation is measured by the increase in UV-Vis absorbance; (B) vesicle formation by rehydration of lipid thin film in the presence of a membrane-soluble dye (naphtho[2,3-α]pyrene). As vesicles are formed, more dye is trapped in bilayers, thus increasing the fluorescence intensity. See Methods for details.

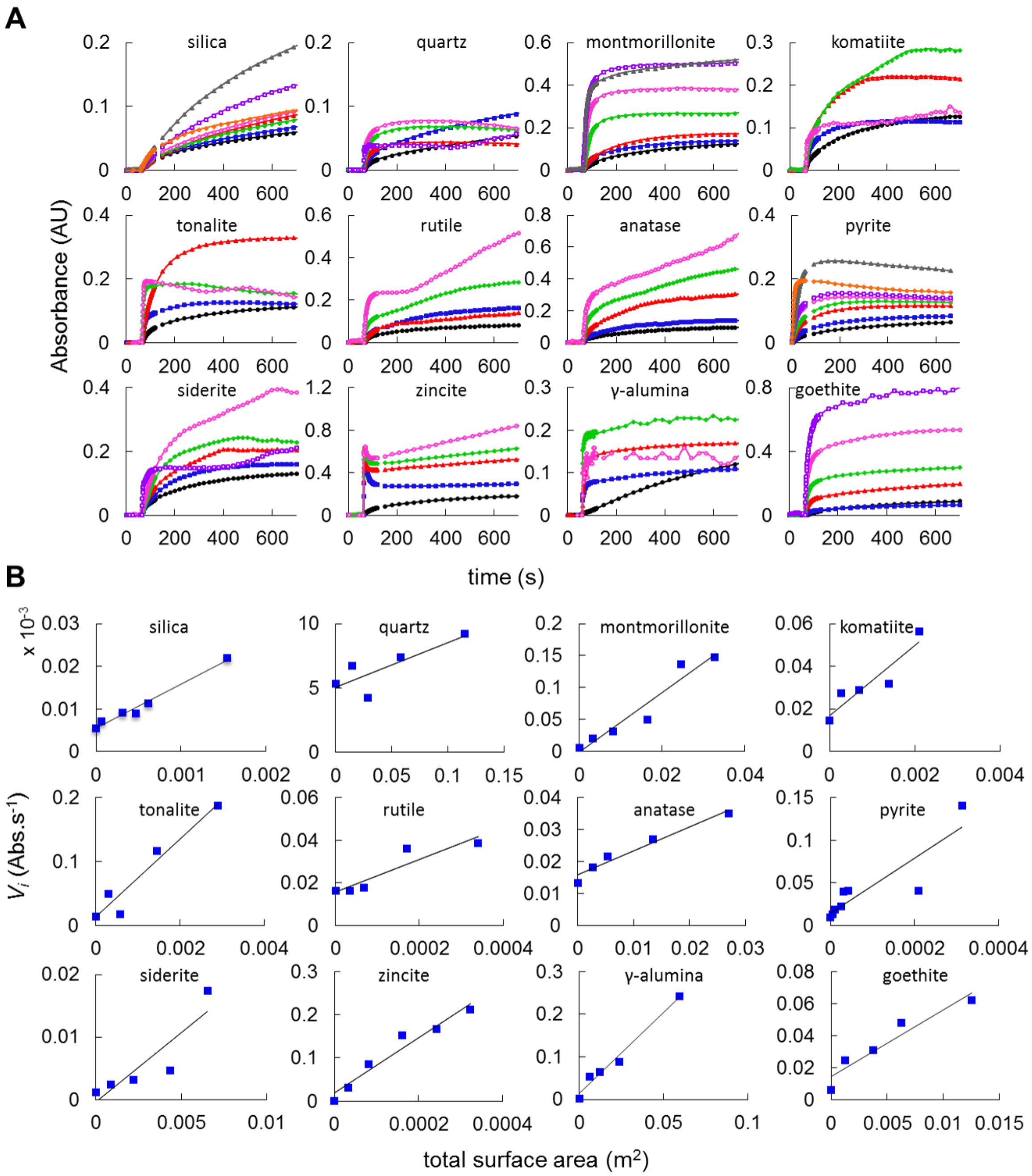

C

Initial rates normalized per unit surface area to yield the parameter, r_o, which is plotted in Figure 2.

Mineral	r_o (Abs.s^{-1}.m^{-2})	STD	Mineral	r_o (Abs.s^{-1}.m^{-2})	STD
silica	0.0345	0.01	anatase	0.7546	0.09
quartz	10.318	0.72	pyrite	322.01	68.8
montmorillonite	4.652	0.63	siderite	2.210	0.66
komatiite	16.450	3.83	zincite	638.967	74.42
tonalite	61.070	9.51	γ-alumina	3.782	0.33
rutile	76.335	18.53	goethite	4.126	0.77

Extended Data Figure 3. Kinetics of DA vesicle formation in the presence of minerals as measured by UV/Vis. (A) Kinetics graphs after subtraction of mineral baselines. The experimental conditions were 30 mM DA, in 200 mM HEPES buffer pH 7.1 ± 0.1 and mineral particle loading ranging from 0.01 to 2 $mg.mL^{-1}$ depending on the particular mineral. Graphs are arranged in order of increasing IEP of minerals; (B) initial rates of vesicle formation as a function of the total surface area; (C) initial rates normalized per unit surface area (r_0). r_0 corresponds to the slope of the linear fit in B and represents an intrinsic parameter of the mineral. r_0 and its standard deviation were calculated using the LINEST function of Excel 2013. See Methods for details.

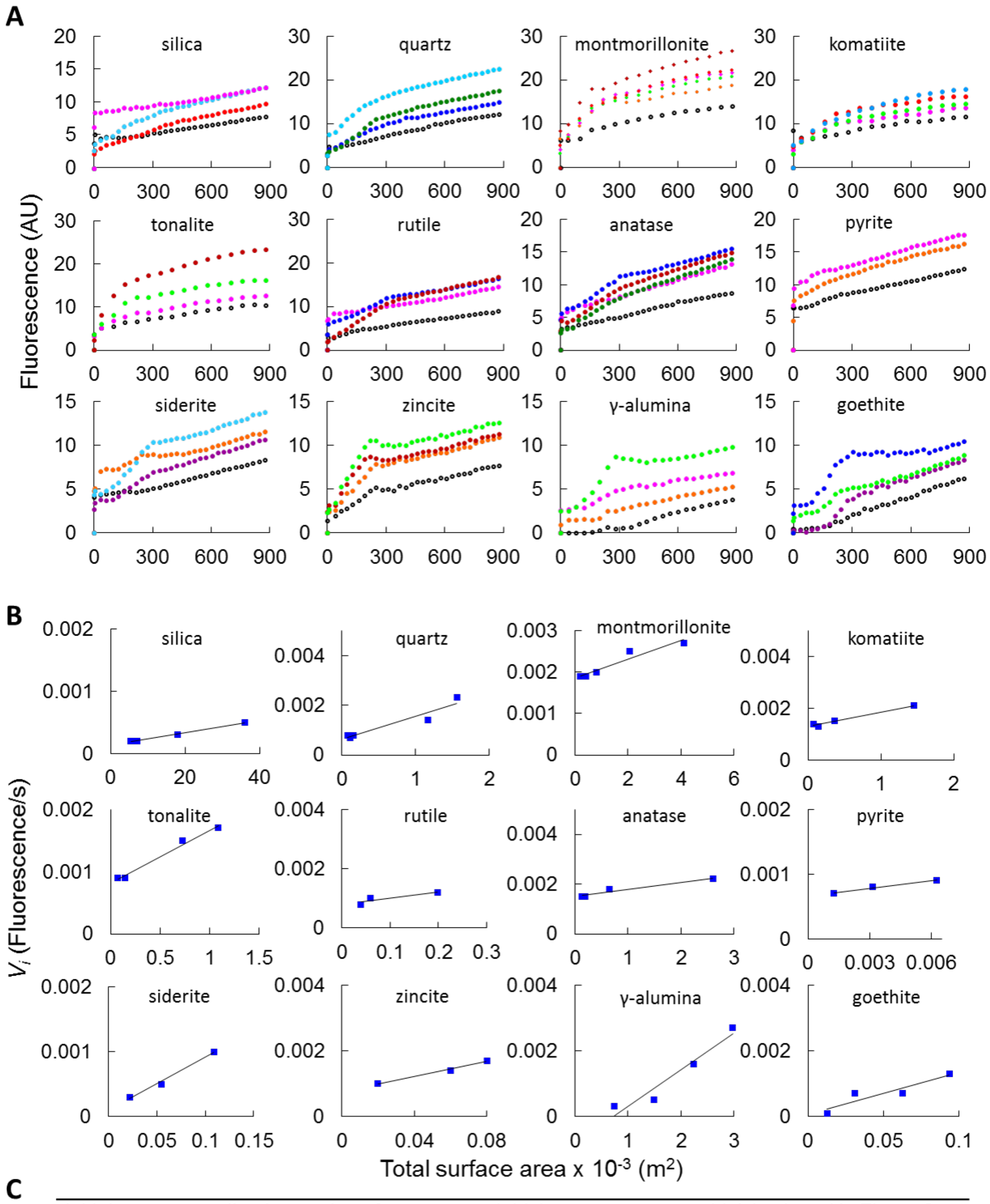

C

Initial rates normalized per unit surface area to yield the parameter, r_o, which is plotted in Figure 2.

Mineral	r_o ($Int.s^{-1}.m^{-2}$)	STD	Mineral	r_o ($Int.s^{-1}.m^{-2}$)	STD
silica	0.01	0.0006	Anatase	0.277	0.05
quartz	0.92	0.16	Pyrite	39.2	5.66
montmorillonite	0.223	0.04	siderite	8.16	0.81
komatiite	0.554	0.06	zincite	11.42	1.24
tonalite	0.844	0.08	γ-alumina	1.12	0.21
rutile	2.132	0.92	goethite	12.73	3.6

Extended Data Figure 4. Kinetics of DA/DOH vesicle formation in the presence of minerals as measured by fluorescence. (A) the experimental conditions were 10 mM DA/DOH (2:1), in 200 mM bicine buffer pH 8.1 ± 0.2 and mineral particle loading ranging from 0.01 to 1 $mg.mL^{-1}$ depending on the particular mineral. Graphs are arranged in order of increasing IEP of minerals; (B) initial rates of vesicle formation as a function of the total surface area; (C) initial rates normalized per unit surface area (r_0). r_0 corresponds to the slope of the fit in B. r_0 and its standard deviation were calculated using the LINEST function of Excel 2013. See Methods for details.

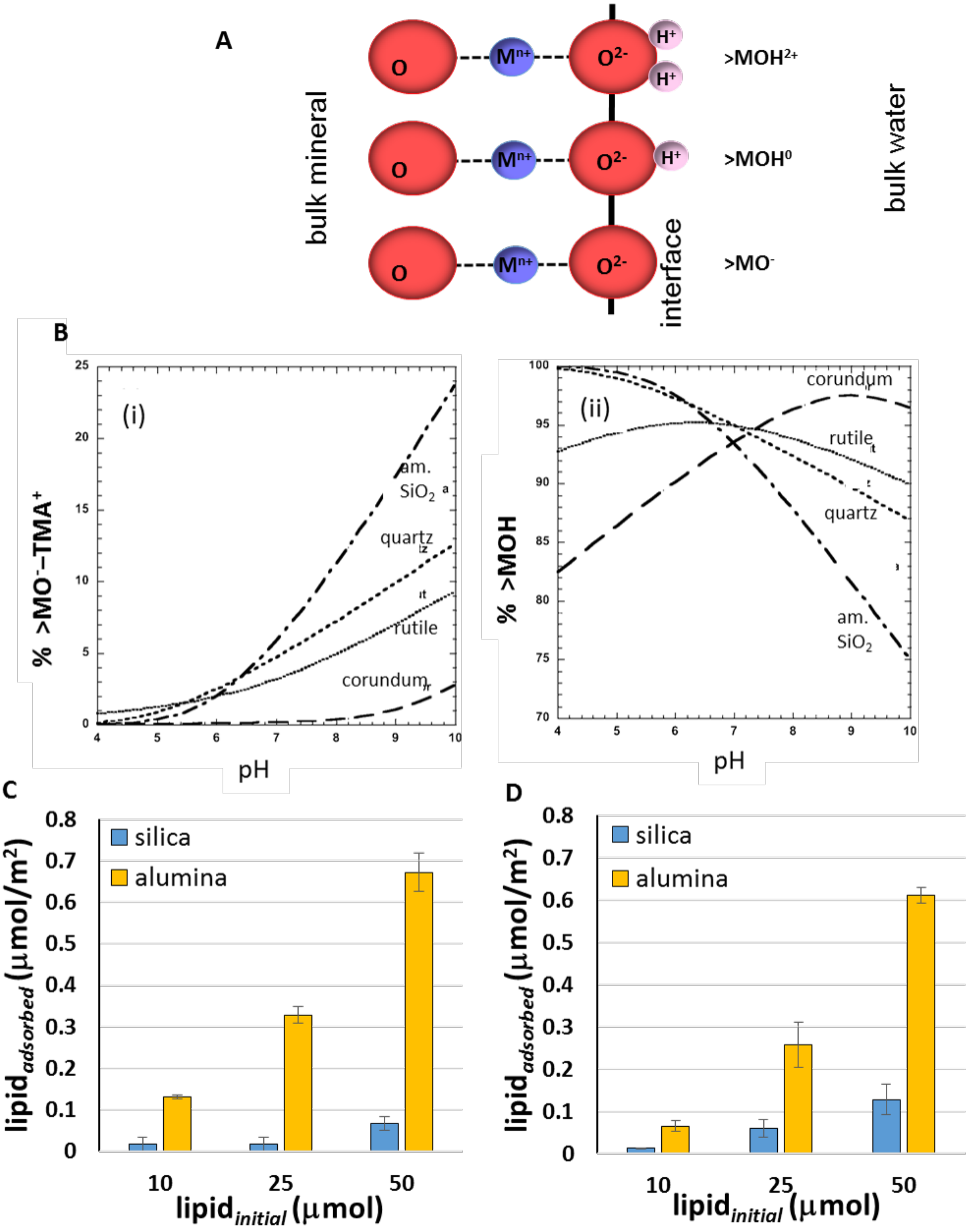

A
bulk mineral
O
M^{n+}
O^{2-}
H^+
$>MOH^{2+}$
$>MOH^0$
$>MO^-$
interface
bulk water
B
(i)
% $>MO^-$–TMA^+
am. SiO_2
quartz
rutile
corundum
pH
(ii)
% >MOH
corundum
rutile
quartz
am. SiO_2
pH
C
lipid$_{adsorbed}$ (μmol/m^2)
silica
alumina
lipid$_{initial}$ (μmol)
D
lipid$_{adsorbed}$ (μmol/m^2)
silica
alumina
lipid$_{initial}$ (μmol)

Extended Data Figure 5. Schematic representation of surface charge on oxides as a result of proton adsorption and desorption at under-coordinated oxygen atoms exposed at the surface. (A) the metal atom, “M,” near the surface, which is bonded to the surface oxygen, nominally contributes one positive charge to the surface oxygen. The oxygen has a double negative charge so the net charge on the surface site is -1 and the site is denoted as $>MO^-$, where “>” indicates that the surface metal is bound to other oxygen atoms below the surface. The adsorption of one proton to such a surface, therefore, results in a neutrally charged site, $>MOH^0$, and adsorption of a second proton (or un-dissociated water molecule) results in a positively charged site, $>MOH_2^+$; (B) the fraction of negatively-charged (i) and neutrally-charged (ii) surface sites as a function of pH, reproduced with permission from Sahai (2002)[34]. In this example, the negative charge is screened by tetramethyl ammonium forming an outer-hydration sphere complex with the negatively-charged surface site. Other examples of counter-ions include Na^+, K^+, or Cs^+. Note that neutrally-charged sites dominate at all pHs. Negatively-charged sites constitute only a small fraction of sites at all pHs; the same holds true for positively-charged surface sites. Thus, ions and other molecules can adsorb by electrostatic interactions at the charged sites as well as by hydrogen bond interactions. For large molecules such as lipids, van der Waals interactions can contribute, additionally, to associate with the surface. (C) and (D) adsorption of DA (pH ~ 7.1) and DA/DOH (pH ~ 8.1), respectively, on silica and alumina gels. Error bars represent standard deviation of triplicate samples. See Methods for details.

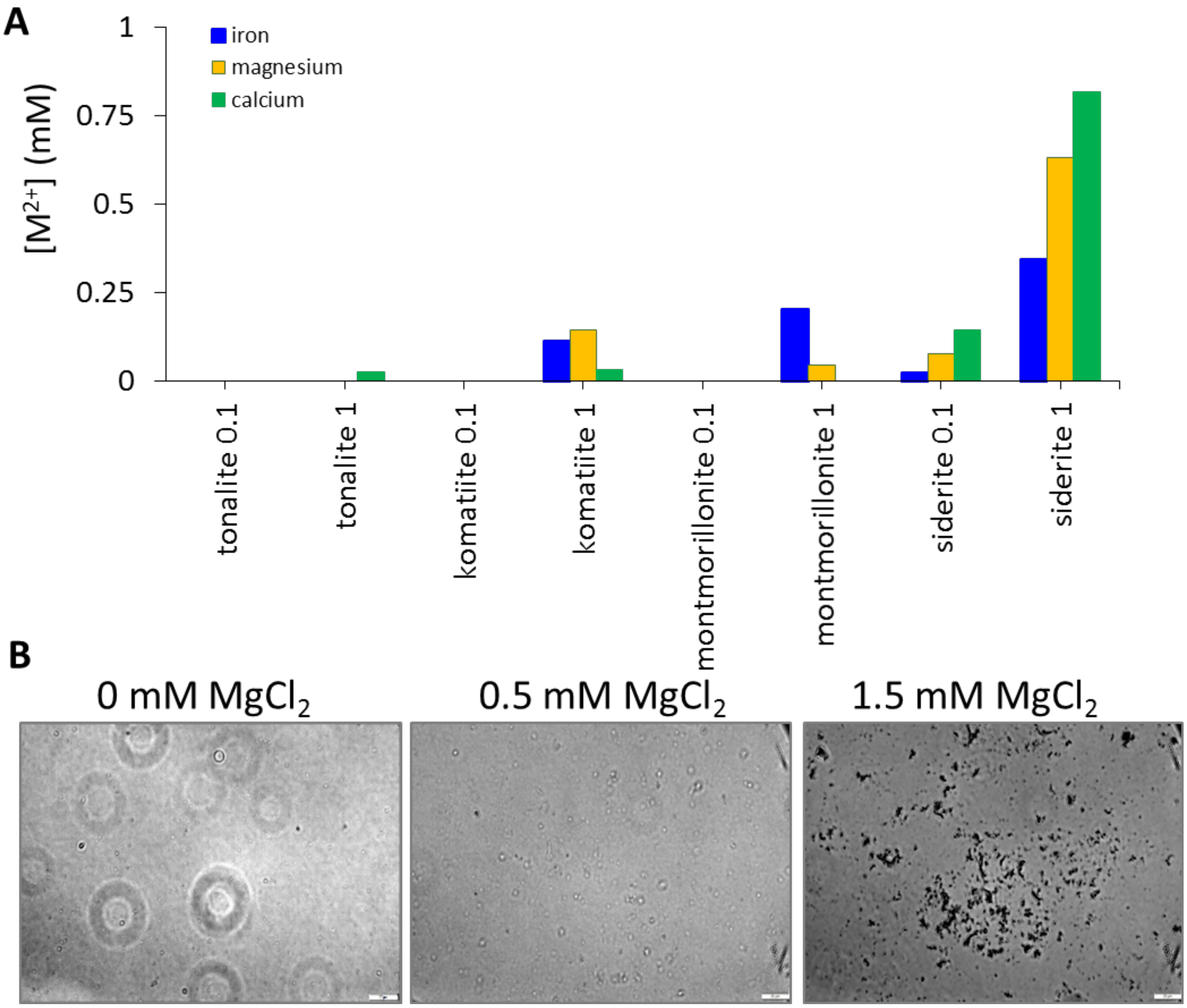

Extended Data Figure 6. Ion-leaching from minerals and effect on DA vesicle stability at pH 7.1. (A) Concentration of ions leached by partial dissolution, as analyzed by ICP-OES. See Methods for details; (B) phase contrast microscopy images of DA (50 mM) in the presence of $MgCl_2$ at pH 7.1 (HEPES, 200 mM). At 0 and 0.5 mM, abundant vesicles are seen. At 1.5 mM, black spots representing DA-Mg^{2+} aggregation, although vesicles are still present. Scale bars represent 20 µm. See Methods for details.

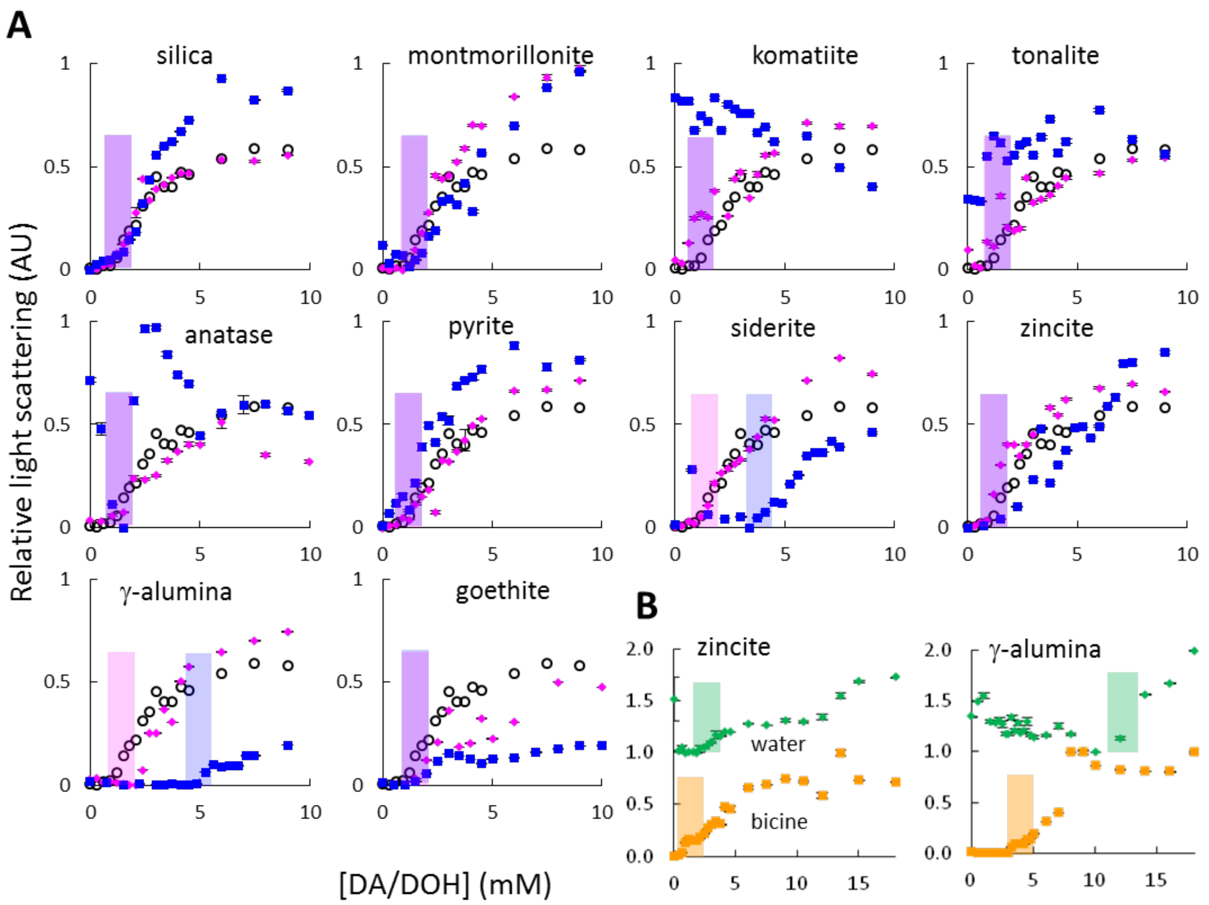

Extended Data Figure 7. CVC of DA/DOH (2:1) in the presence of minerals as determined by DLS at pH 8.1 (bicine, 200 mM). (A) the graphs are arranged in order of increasing IEP. Black, pink and blue symbols correspond to the no-mineral control system, 0.1 and 1 mg.mL^{-1} particle loadings, respectively. The pink and purple bars indicate the approximate range of CVC values marked by the sharp increase in light scattering intensity. Little or no effect of minerals was observed except in the case of siderite, alumina and goethite at loading of 1 mg.mL^{-1}; (B) effect of buffer. CVC was measured in ultrapure water in the presence of the positively-charged minerals at 1 mg.mL^{-1} loading. Orange and green symbols correspond, respectively, to bicine solution and water. Orange and green bars represent the approximate range of CVCs. The

effect of mineral-IEP on CVC (increase) is more noticeable in water. Error bars represent standard deviation of at least duplicate experiments; triplicate measurements were made for each sample in each experiment. See Methods for details.

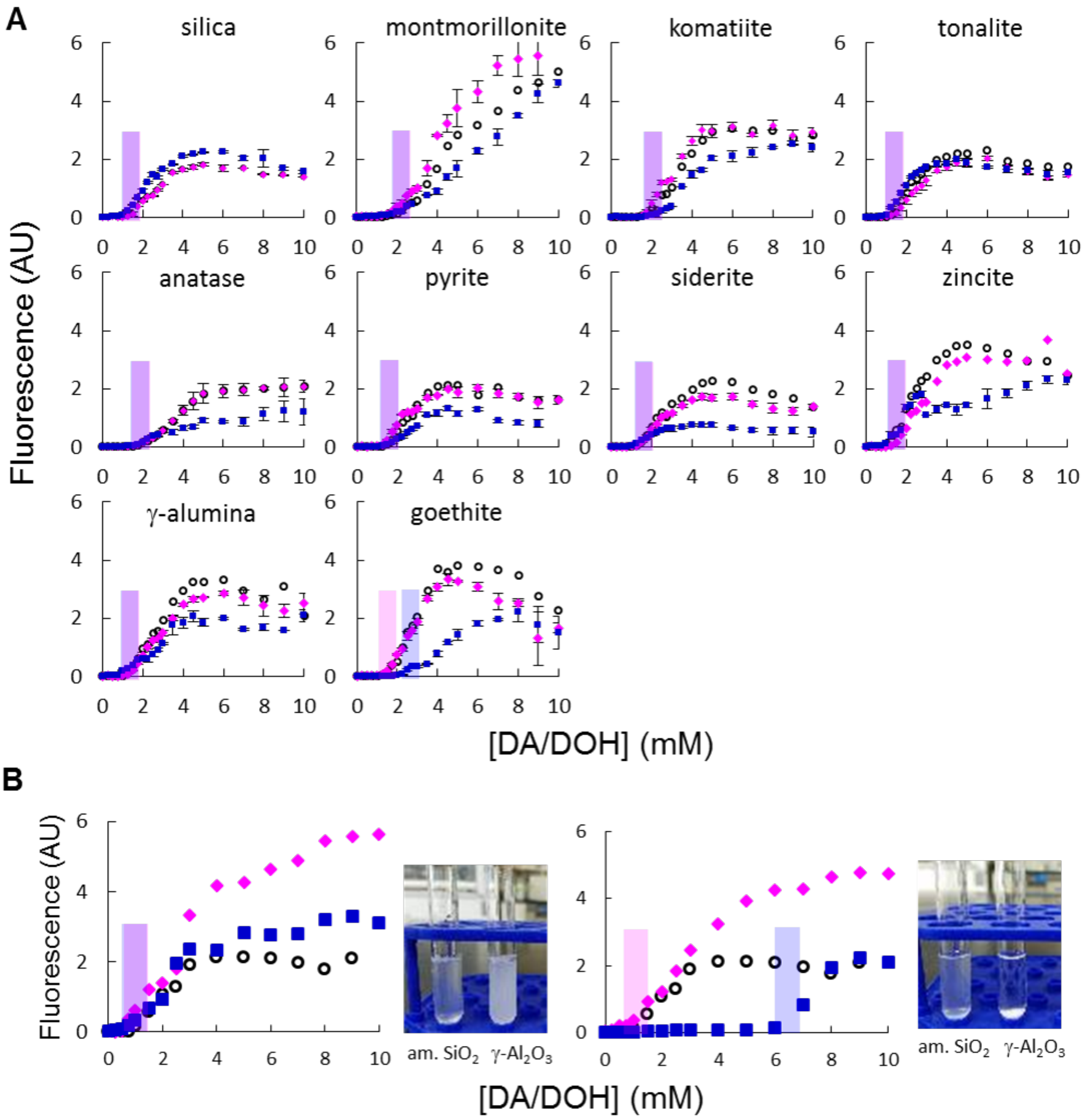

Extended Data Figure 8. CVC of DA/DOH (2:1) in the presence of minerals as determined by fluorescence at pH 8.1 ± 0.2 (bicine, 200 mM). (A) the graphs are arranged in order of increasing IEP. Black, pink and blue symbols correspond to no-mineral control, 0.1 $mg.mL^{-1}$ and 1 $mg.mL^{-1}$ particle loadings, respectively. Error bars represent standard deviation of triplicate samples.

The pink and purple bars indicate the approximate range of CVC values marked by the sharp increase fluorescence. Little or no effect of minerals was observed except in the case of the positively-charged mineral, goethite, at a loading of 1 $mg.mL^{-1}$; (B) effect of mineral settling at 1 $mg.mL^{-1}$. Black, pink and blue symbols correspond to no-mineral control, silica and alumina, respectively. Left and right graphs correspond to measurements at time 0 h and 2 hours after mixing, respectively. See Methods for details.

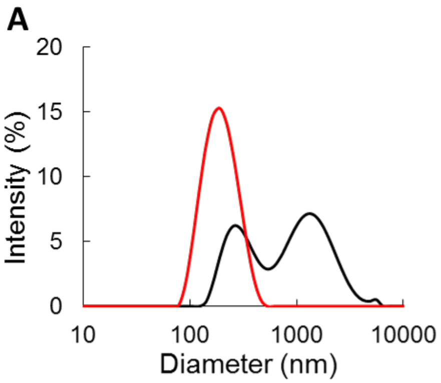

	PDI	diameter, Z-average (nm)	intercept
unextruded	0.451	568.9	0.898
extruded	0.103	179.5	0.92

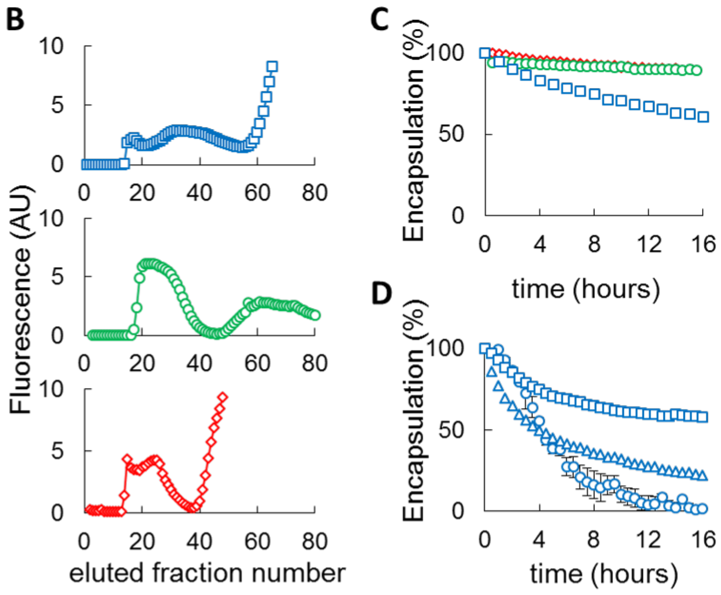

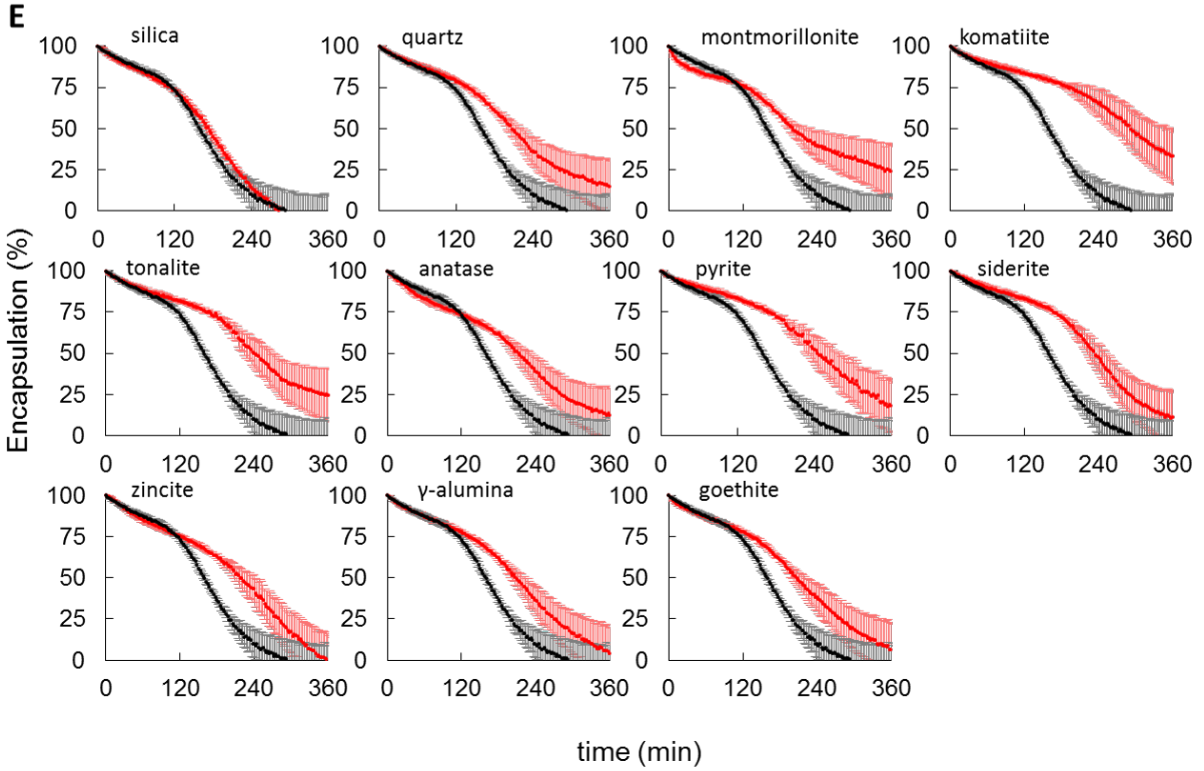

Extended Data Figure 9. Calcein leakage assay at pH 8 ± 0.1. (A) Typical scattering intensity of un-extruded (black) and 200 nm-extruded (red) vesicles as a function of the average diameters; (B) column purification of calcein-loaded vesicles. Top, DA/DOH; middle, OA; and bottom, POPC; (C) calcein leakage from different lipid systems. DA/DOH (25 mM), blue squares; OA (7 mM), green circles; and POPC (1 mM), red diamonds; (D) calcein leakage from different DA/DOH concentrations. 25 mM, squares; 10 mM, triangles; and 7 mM, circles. Error bars represent standard deviation of triplicate samples; (E) calcein leakage from DA/DOH vesicles at 7 mM final lipid concentration. Black and red curves correspond to no-mineral control and 0.1 $mg.mL^{-1}$ particle loading, respectively. Error bars represent standard deviation of triplicate samples. See Methods for details.
